# Droplet Microfluidics Approach for Single-DNA Molecule Amplification and Condensation into DNA-Magnesium-Pyrophosphate Particles

**DOI:** 10.3390/mi8020062

**Published:** 2017-02-20

**Authors:** Greta Zubaite, Karolis Simutis, Robertas Galinis, Valdemaras Milkus, Vaidotas Kiseliovas, Linas Mazutis

**Affiliations:** Institute of Biotechnology, Vilnius University, Saulėtekio Avenue 7, LT-02571 Vilnius, Lithuania; greta.zubaite@gmail.com (G.Z.); karolis.simutis@gmail.com (K.S.); robertas.galinis@bti.vu.lt (R.G.); valdemaras.milkus@gmail.com (V.M.); vaidotas.kiseliovas@bti.vu.lt (V.K.)

**Keywords:** in vitro expression, DNA condensation, droplet microfluidics, phi29 DNA polymerase

## Abstract

Protein expression in vitro has broad applications in directed evolution, synthetic biology, proteomics and drug screening. However, most of the in vitro expression systems rely on relatively high DNA template concentrations to obtain sufficient amounts of proteins, making it harder to perform in vitro screens on gene libraries. Here, we report a technique for the generation of condensed DNA particles that can serve as efficient templates for in vitro gene expression. We apply droplet microfluidics to encapsulate single-DNA molecules in 3-picoliter (pL) volume droplets and convert them into 1 μm-sized DNA particles by the multiple displacement amplification reaction driven by phi29 DNA polymerase. In the presence of magnesium ions and inorganic pyrophosphate, the amplified DNA condensed into the crystalline-like particles, making it possible to purify them from the reaction mix by simple centrifugation. Using purified DNA particles, we performed an in vitro transcription-translation reaction and successfully expressed complex enzyme β-galactosidase in droplets and in the 384-well format. The yield of protein obtained from DNA particles was significantly higher than from the corresponding amount of free DNA templates, thus opening new possibilities for high throughput screening applications.

## 1. Introduction

Droplet microfluidics offers a powerful tool to isolate, amplify and quantify nucleic acid molecules in a massively parallel manner [1,2]. For biological assays in particular, the droplet format provides not only significant savings for the cost of the reagents, but also increased analytical sensitivity and ultra-high throughput capabilities [3,4]. These features have led to a growing number of applications of droplet microfluidics technology for DNA amplification and detection [5,6,7], library preparation [8,9], in vivo directed evolution [10,11,12], single-cell assays [13,14,15] and others [16,17,18]. Droplet-based approaches also have shown promising results for screening protein crystallization conditions [19] and for studying nucleation events during amyloid aggregation [20]. Compartmentalization of reagents into microscopic droplets can shift reaction equilibrium towards product formation and as a result improve the efficacy of chemical reactions [21]. As was shown recently, in the case of the DNA isothermal amplification reaction, the compartmentalization enhances amplified nucleic acid condensation into crystalline-like structures [22]. It was shown that during the multiple displacement amplification (MDA) reaction, the magnesium ions from the buffer and DNA replication reaction byproduct inorganic pyrophosphate trigger amplified DNA condensation into DNA-magnesium-inorganic pyrophosphate (DNA-Mg-PP_i_) particles. These particles can be purified from the reaction mix preserving their compact structure and serve as a template for an improved protein synthesis in vitro [21]. However, the conditions that affect DNA-Mg-PP_i_ particle’s size have not been fully understood. Moreover, the purification of DNA-Mg-PP_i_ particles from the MDA reaction mix using preparative agarose gel electrophoresis followed by centrifugation has led to significant losses of starting material, which could limit broader applications. 

Herein, we extend our previous studies and report generation of ~1300 nm-sized DNA-Mg-PP_i_ particles that retain significantly higher amounts of clonally-amplified DNA and afford the in vitro expression of multi-subunit enzymes, such as β-galactosidase. We describe an improved approach for performing single DNA molecule amplification and condensation into micrometer-sized particles and their use for protein synthesis in vitro. The purification procedure described here recovers ~60%–90% of DNA particles in comparison to ~0.5%–1.0% from the previous work [21]. The purified particles can be re-encapsulated and serve as a template for the in vitro transcription-translation (IVTT) reaction in microdroplets. In addition, we found that the heat-inactivation step, which is typically used to stop the MDA reaction, actually breaks DNA particles into smaller, ~200 nm-sized globular structures. Finally, we measured the catalytic activity of in vitro synthesized protein in a 384-well plate and found that the IVTT reaction supplemented with ~5 × 10^4^ DNA particles produces similar yields of functional enzyme, as many as ~10^9^–10^10^ molecules of native DNA template. 

The approach reported here, in principle, could be adapted to a variety of proteins or enzymes when their in vivo expression is inefficient or incompatible with living functions. It is important to emphasize that single DNA molecule isolation, amplification and condensation into particles inside droplets prevents the newly-synthesized DNA from forming catenated structures with DNA particles from other droplets, which is an important consideration when preparing clonally-amplified gene libraries. Since purified DNA particles retain sufficiently large numbers of functional gene copies (~10^5^ copies per particle), it leads to a considerable improvement of the IVTT reaction of complex proteins. The reported technique should benefit biological applications relying on completely in vitro assays, such as directed evolution or drug and enzyme screening.

We postulate that the formation of crystalline-like DNA-Mg-PP_i_ structures inside droplets is defined by the phase transition from a dissolved (disordered) state into an ordered state [23]. For the nucleic acids, such a transition is typically enhanced in the presence of positively-charged agents (cations, polymers or electrostatic surfaces) that neutralize the negative charge of the phosphodiester bond and facilitate DNA condensation [24]. Interestingly, in the case of the DNA amplification reaction by DNA polymerase, the DNA incorporation into crystalline-like particles occurs during the accumulation of a reaction byproduct, inorganic pyrophosphate (PP_i_), which is negatively charged [22]. It is well known that in the presence of magnesium ions, the PP_i_ precipitates into insoluble and heat-resistant Mg_2_(PP_i_) spherulitic particles [25,26,27]. In our case, the inorganic Mg_2_(PP_i_) particle may act as a substrate for the adsorption of newly-synthesized DNA strands. The DNA condensation theory [24] suggests that magnesium ions chelated by inorganic pyrophosphate should decrease the electrostatic repulsion between the newly-synthesized DNA strands, thereby facilitating self-assembly into crystalline-like structures. When the DNA amplification reaction is confined in a small volume, the conditions for DNA condensation become quickly satisfied because of continuous accumulation of reaction products: the newly-synthesized DNA strands and magnesium-pyrophosphate crystals. The resulting DNA-Mg-PP_i_ particles can be purified from the reaction mix and still retain significant amounts of clonally-amplified DNA, thus offering attractive possibilities for biomedical and biochemical applications.

## 2. Materials and Methods

### 2.1. Microfluidic Device Fabrication and Operation

Polydimethylsiloxane (PDMS)-glass microfluidics devices having rectangular microfluidic channels 10 or 20 μm deep were obtained from Droplet Genomics. Sterile 1-mL syringes (Braun, Bad Arolsen Germany) mounted on syringe pumps (Harvard Apparatus, Holliston, MA, USA) and connected to the microfluidics device via 27 G needle and 0.56 mm × 1.07 mm polytetrafluoroethylene (PTFE) tubing were used to infuse fluids into the microfluidics chip. Three-picoliter droplets were generated by adjusting flow rates to 100 μL/h for the reaction mix and 300 μL/h for the continuous phase. The droplet stabilization oil (Droplet Genomics, Vilnius, Lithuania) was used as the continuous phase to stabilize droplets against coalescence. During encapsulation, samples were kept at 4 °C using an ice-cold jacket and collected in the form of an emulsion in a 0.2-mL polymerase chain reaction (PCR) tube (Eppendorf, Hamburg, Germany) prefilled with 80 μL of mineral oil (Sigma, St. Louis, MO, USA) and placed in anice-rack (Eppendorf). 

### 2.2. Single-DNA Molecule Encapsulation and Amplification

The MDA reaction mix contained pIVEX2.2-lacZ-his plasmid, 1× phi29 reaction buffer (33 mM Tris-acetate (pH 7.9), 10 mM Mg-acetate, 66 mM K-acetate, 0.1% (*v*/*v*) Tween 20, 1 mM dithiothreitol (DTT)), 50 μM exo-nuclease resistant hexanucleotide primers, 1 mM of each deoxynucleoside triphosphates (dNTP), 0.4% (*w*/*v*) Pluronic F-127 and 0.8 U/μL phi29 DNA polymerase (Thermo Fisher Scientific, Vilnius, Lithuania). The reaction components were mixed in DNA LoBind tubes (Eppendorf) by adding DNA template, nuclease-free water, Pluronic F-127 and hexamers and then heated to 90 °C for 20 s to allow primer annealing. Next, the mixture was quickly transferred onto ice and, following the addition of the remaining components, encapsulated using the microfluidic device. Fluids were injected into the microfluidics device at 100 μL/h for the aqueous phase and 300 μL/h for the carrier oil. The encapsulation step typically took ~60 min and generated ~10^7^ monodisperse 3-pL droplets, which were collected off-chip in the form of an emulsion, incubated for 16–18 h at 30 °C and heated at 65 °C for 10 min to inactivate phi29 DNA polymerase. 

### 2.3. Staining of Droplets and Fluorescence Analysis

The droplets were stained with SYBR Green dye I (Life Tech, Rockland, ME, USA) by adding 2 μL of 100× dye solution to 2 μL of droplet stabilization oil. The dye passively migrates between the droplets and stains dsDNA. Fluorescence images were recorded with a 1.5 megapixel digital camera (Ds-Qi1, Nikon, Tokyo, Japan) on an inverted microscope (Nikon Ti-U Eclipse). Fluorescence excitation was set at 470 ± 20 nm (with a 100-ms exposure) and emitted light collected at 525 ± 25 nm. Recorded images were processed with open-source software Fiji [28] to count the total number of droplets, the number of fluorescent droplets, mean fluorescence intensity values and the coefficient of variation.

### 2.4. Transmission Electron Microscopy Imaging

Transmission electron microscopy images were recorded on a FEI Morgagni 268 instrument (FEI Company, ‎Hillsboro‎, ‎OR, USA). A 4-μL sample was placed on a grid (100 Formvar/Carbon Films, Cu 400 mesh, QUANTIFOIL, Großlöbichau, Germany) and incubated for 1 min at room temperature. After draining excess liquid, the sample was washed twice with Milli-Q purified water, stained with 2% (*w*/*v*) uranyl acetate for 20 s and then imaged. 

### 2.5. DNA Particle Purification

After the DNA amplification reaction, samples were digested with restriction endonuclease (REase) for 15 min at 37 °C to remove the loose DNA that has not been incorporated into the DNA particles. The RE was chosen such that it would cleave the plasmid once and outside the encoded gene. Then, 1 U/μL of HindIII was used to cleave DNA particles generated from pIVEX2.2-lacZ-his plasmid. Following digestion by REase, the DNA particles (100 μL) were washed with 400 μL of nuclease-free distilled water and centrifuged for 10 min at 10,000 rpm at room temperature. The supernatant was removed, and the DNA particle pellet was re-suspended in 400 μL of nuclease-free distilled water. This was followed by two additional washes after which the DNA particle pellet was re-suspended in a final volume of 50 μL nuclease-free distilled water. The purified DNA particles were then stained with 10× SYBR Green I dye, loaded onto a hemocytometer, imaged under fluorescence microscope and counted using Fiji software. Purified DNA particles were stored at 4 °C.

### 2.6. Coupled In Vitro Transcription and Translation

β-galactosidase enzyme expression was performed using an IVTT system purchased from New England Biolabs (PURExpress^®^ in vitro Protein Synthesis Kit, NEB, Ipswich, MA, USA) in the presence of RNase inhibitor Ribolock (Thermo Fisher Scientific, Vilnius, Lithuania). 

IVTT reaction in 384-well format: The in vitro expression of lacZ in a 384-well format (10 μL/well) was performed by preparing two sets of IVTT reaction mixtures. The first set of reactions was supplemented with 0.5 to 500 ng of pIVEX2.2-lacZ-his plasmid, which translated into 10^11^–10^8^ copies of free DNA molecules per 10 μL. The second set of reactions contained purified DNA particles diluted down to 46,000, 23,000 and 5000 particles per well. Reactions were then incubated at 37 °C for 3 h to allow gene expression to occur. The catalytic activity of in vitro synthesized lacZ enzyme was recorded by mixing 1 μL of IVTT mix with 9 μL of 1× phi29 buffer (NEB) supplemented with 1 μM fluorescein-di-β-d-galactopyranoside (FDG). The fluorescence signal was measured in a 384-well microtiter plate (polypropylene, black, flat, clear-bottom, Corning Inc., Corning, NY, USA) using a Synergy H4 plate reader set at 488 nm × 20 nm excitation and 530 nm × 20 nm emission wavelengths (gain 75 and 50).

IVTT reaction in droplets: Purified DNA particles stained with ethidium bromide (15 μg/mL) were mixed with the IVTT solution containing 1 μM of β-galactosidase substrate FDG and encapsulated in 18-pL droplets using the 20-μm microfluidics device. The flow rates for IVTT reaction encapsulation were 100 μL/h for the aqueous phase and 250 μL/h for the carrier oil. The encapsulation time typically took ~20 min, at 4 °C. Protein synthesis in vitro was evaluated after incubating the emulsion at 37 °C for 1 h. The fluorescence was recorded using an excitation wavelength at 475 nm × 12 nm and an emission wavelength at 559 nm × 17 nm. 

## 3. Results and Discussion

### 3.1. Single DNA Molecule Encapsulation and Amplification

Droplet microfluidics has opened new analytical possibilities for biological sciences, diagnostics and biomedicine [29]. Single-DNA molecule isolation, amplification and digital quantification have gained broad interest for the evaluation of rare diseases [30,31,32], quantifying the absolute number of nucleic acids in a sample [33,34] or disease diagnostics [35,36]. In these and others examples, the sample containing diluted suspension of nucleic acids is encapsulated in microfluidic droplets and amplified using the PCR reaction followed by fluorescence readout. However, the template size in most of the reports is in the range of ~200–300 nucleotides, which prevents their use for other important applications, such as directed evolution or drug screening. In addition, droplets can experience coalescence during thermocycling [34], thus imposing certain limitations on the experimental design. In this context, DNA amplification driven by phi29 DNA polymerase provides an alternative approach to amplify long DNA molecules and because of isothermal reaction conditions [37,38], the potential problems associated with emulsion stability become irrelevant. Moreover, the ability not only to amplify individual DNA molecules, but also to express genes from the amplified template opens many interesting possibilities for high throughput screening applications.

**Figure 1:**
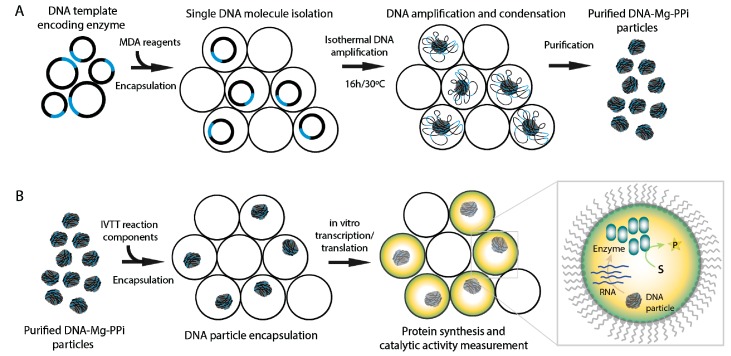
Schematics of the experimental platform. (**A**) Single-DNA molecule amplification. DNA template encoding an enzyme is encapsulated in microfluidic droplets and converted into condensed DNA-magnesium-inorganic pyrophosphate (DNA-Mg-PP_i_) particles during the multiple displacement amplification (MDA) reaction. After the MDA step, the resulting DNA particles are released, briefly digested with endonuclease and separated by centrifugation. (**B**) The in vitro transcription-translation (IVTT) reaction using purified DNA particles as a template. DNA particles are encapsulated with IVTT components to initiate protein synthesis in vitro in droplets. The catalytic activity of translated enzyme is then measured by fluorescence readout.

**Figure 2:**
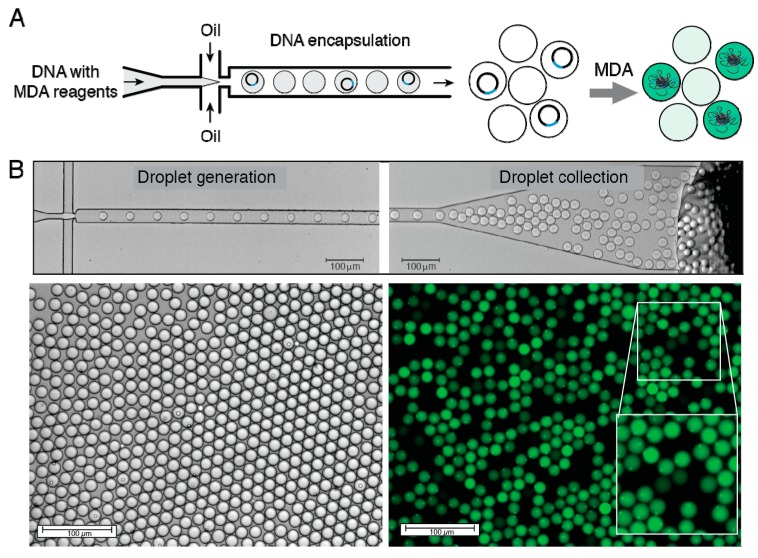
DNA encapsulation and amplification using droplet microfluidics. (**A**) Schematic representation of single-DNA molecule isolation and condensation into DNA particles. (**B**) Top: still images showing droplet generation and collection. Bottom: the bright field and fluorescence micrographs showing emulsion droplets after MDA reaction. The bright fluorescent objects are visible in droplets. Scale bars, 100 μm.

The overall experimental strategy of this work is summarized in Figure 1. We first emulsified 3.6 kb circular nucleic acid molecules and multiple displacement amplification (MDA) reagents into 3-pL droplets using 10 μm-deep microfluidics device. The MDA reaction mix contained DNA template (pIVEX2.2-lacZ-his plasmid encoding enzyme β-galactosidase), phi29 DNA polymerase, exo-resistant oligonucleotides and other reagents needed for efficient isothermal DNA amplification reaction (see the Materials and Methods). We used diluted DNA concentrations (0.63 pM) so that one droplet would contain one molecule on average (λ ~ 1.0). The portioning of DNA molecules into droplets follows the Poisson equation, $P(X=k)=\frac{e^{-\lambda }}{k!}\lambda^{k}$, which describes the probability *P* of having *k* DNA molecules per droplet (*k* = 0, 1, 2…), where λ is the average DNA molecule number per droplet. Using the reaction mix containing 0.63 pM DNA, the Poisson equation predicts 36.7% of 3-pL droplets being empty, 36.7% having one and 26.6% having ≥2 DNA molecules. High droplet generation speed (~4000 s^−1^) allowed the collection of over 10^7^ droplets in the form of an emulsion in less than 1 h. Droplets loaded with MDA reaction mix were collected off-chip into a collection tube and incubated for 16 h at 30 °C to initiate the isothermal DNA amplification reaction by phi29 DNA polymerase. After, off-chip incubation droplets were heated for 10 min at 65 °C and stained with SYBR Green I dye, which passively migrates between the droplets and becomes fluorescent upon binding dsDNA (Figure 2). In agreement with our previous studies [22], the amplified DNA formed condensed particles clearly visible under the fluorescent microscope (Figure 2B, inset). 

**Figure 3:**
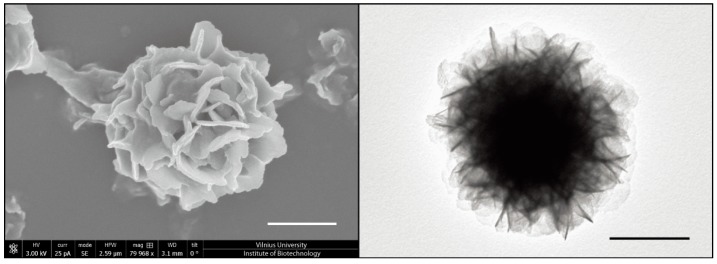
Electron microscopy analysis of DNA-magnesium-pyrophosphate particles produced during the multiple displacement amplification reaction. Scanning electron microscopy (**A**) and transmission electron microscopy (**B**) images of a DNA-Mg-PP_i_ particle produced in a 3-pL droplet during the MDA reaction (excluding the heat-inactivation step at 65 °C). To reveal the petal-like structure of a DNA particle, the transmission electron microscopy (TEM) image was recorded without staining with uranyl acetate. Scale bars, 500 nm.

**Figure 4:**
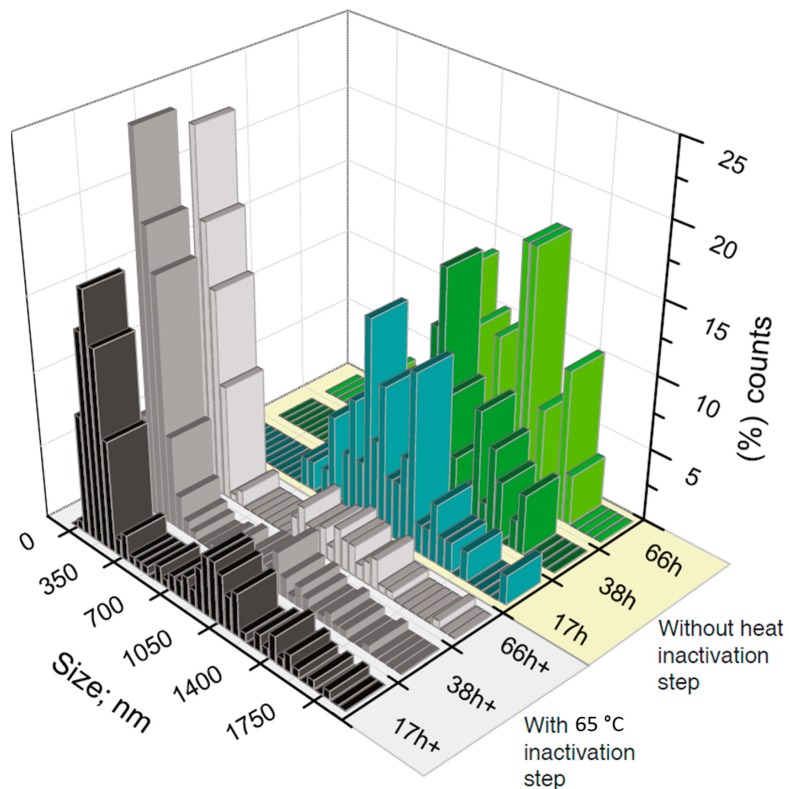
Size distribution of DNA-magnesium-pyrophosphate particles as a function of multiple displacement amplification reaction times, with and without the heat-inactivation step. At different time points (17, 38 and 66 h) of the MDA reaction in 3-pL droplets at 30 °C, the resulting DNA-Mg-PP_i_ particles were released from droplets and imaged under TEM to measure their size. The first set of measurements (green) was made without heat-inactivation step. The second set of measurements (grey) was made with heat-inactivation step at 65 °C for 15 min that is typically used to inactivate phi29 DNA polymerase and terminate the MDA reaction.

**Figure 5:**
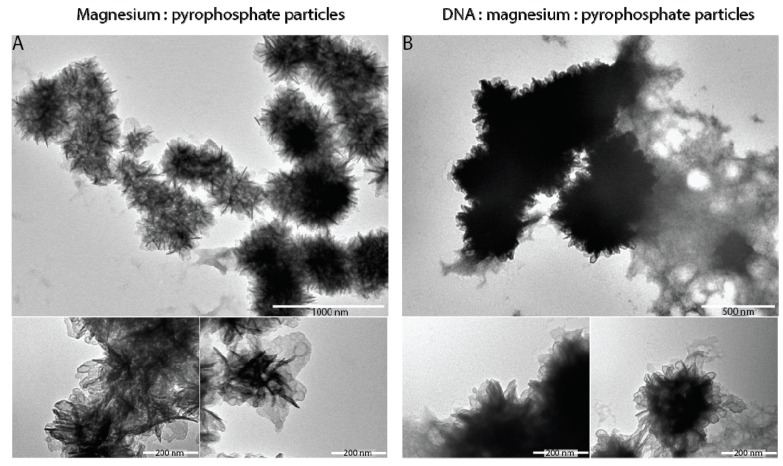
Transmission electron microscopy images of magnesium-pyrophosphate and DNA-magnesium-pyrophosphate particles produced in bulk by mixing individual components. (**A**) Inorganic magnesium-pyrophosphate particles produced by mixing 10 mM MgCl_2_ and 5 mM sodium pyrophosphate (Na₄P₂O₇) in deionized water. (**B**) Particles from (A) mixed with 56 nM plasmid DNA (300 ng/μL) and imaged under TEM. Denser staining with uranyl acetate suggests DNA adsorption. No DNA amplification was involved. The particles in (B) stained with DNA dye (SYBR Green I) became fluorescent, whereas the particles in (A) remained dark. Both types of particles were resistant to treatment by yeast enzyme inorganic pyrophosphatase.

The transmission electron microscopy (TEM) and scanning electron microscopy (SEM) analysis revealed densely packed globular shape particles having the average size of 1233 ± 266 nm (Figure 3). Since the amount of amplified DNA during the MDA reaction increases geometrically over time [37], it would be reasonable to assume that longer incubations should increase the size of DNA particles. However, extended incubations have not affected the DNA nanoparticle’s size, suggesting that MDA reaction in 3-pL droplets has been largely completed in the 16-h window (Figure 4). Similar studies have shown that the yield of amplified DNA in droplets is limited by the available dNTPs and primers [39]. In addition, it appears that the 65 °C heat-inactivation step, which terminates the MDA reaction by denaturating phi29 DNA polymerase, causes DNA particles to break into smaller pieces, as witnessed by the appearance of two particle subpopulations (Figure 4). We further investigated the external features of the DNA particles by TEM and compared them to the inorganic Mg-PP_i_ particles lacking DNA template. To produce inorganic Mg-PP_i_ particles, we mixed 10 mM MgCl_2_ and 5 mM sodium pyrophosphate (Na₄P₂O₇) in deionized water, heated the reaction mix for 5 min at 70 °C and, after cooling down to room temperature (21 °C), immediately imaged the sample under TEM. The TEM analysis revealed spiky spherulites of approximately ~200 nm in size (Figure 5), resembling calcium orthophosphate cements [40]. When the same Mg-PP_i_ particles were mixed with 56 nM of plasmid DNA (~300 ng/μL), the spiky features turned into the petal-like structures closely resembling those DNA particles that were synthesized during the MDA reaction (see Figure 3). As expected, staining the latter particles with DNA stain (SYBR Green I dye) produced a highly fluorescent signal, whereas Mg-PP_i_ particles that were not incubated with DNA remained completely dark. Altogether, our results suggest that inorganic Mg-PP_i_ particles adsorb DNA and, therefore, may act as a seed for DNA assembly into condensed macromolecular structures during the MDA reaction. In support of this notion, Shopsowitz et al. showed that RNA-Mg-PP_i_ particles produced during rolling circle transcription can be stripped off the surface-bound RNA to retain the inorganic Mg_2_P_2_O_7_·3.5H_2_O scaffold core [26]. The fraction of RNA per single particle was found to be around 20 wt %, which we anticipate to be of a similar level for the DNA-Mg-PP_i_ particles produced in this work. Others have also shown the generation of various macromolecular nucleic acid structures that are likely to be mediated through multiple ion bridges coordinated by magnesium ions and pyrophosphate [41,42,43,44,45]. Given our results and previous reports, it is appealing to think that under certain environmental conditions, the inorganic pyrophosphate and divalent ions (magnesium, calcium, etc.) complexes may also induce genomic DNA condensation in cells. Interestingly, earlier research has described bacterial chromosomal DNA condensation (crystallization) induced by negatively-charged Dps proteins and divalent cations [46,47], suggesting that inorganic pyrophosphate possibly could mimic the function of the Dps protein. 

### 3.2. Biological Functionality of Condensed DNA Particles

**Figure 6:**
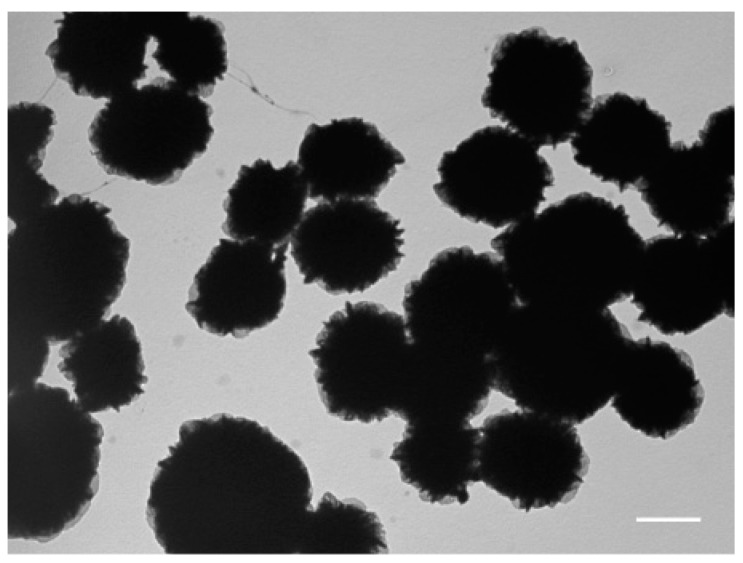
TEM image of purified DNA-magnesium-pyrophosphate particles. DNA particles produced during the MDA reaction were released from droplets, digested with diluted restriction endonuclease enzyme and purified by centrifugation followed by TEM imaging. Scale bar, 1 μm.

**Figure 7:**
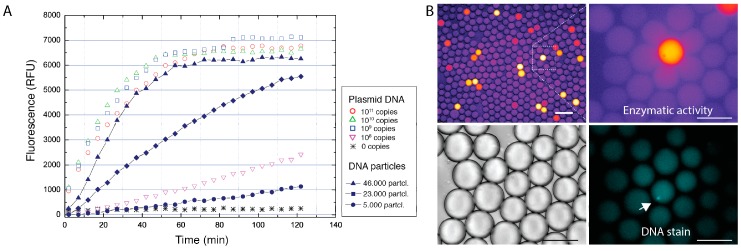
LacZ expression in vitro using purified DNA-magnesium-pyrophosphate particles as a template. (**A**) Evaluation of the catalytic activity of the in vitro synthesized β-galactosidase enzyme. Protein synthesis was performed in a 384-well format using different dilutions of pIVEX-lacZ-his plasmid (10^11^–10^8^ copies in a 10-μL IVTT reaction) and purified DNA-Mg-PP_i_ particles (46,000, 23,000 and 5000 particles in a 10-μL IVTT reaction). The corresponding amount of plasmid DNA and DNA particles is indicated on the right side of the graph. Solid symbols indicate the enzymatic activity of lacZ protein synthesized from the DNA particles, whereas open symbols indicate protein synthesized from the plasmid DNA. (**B**) Evaluation of the IVTT reaction and enzymatic activity in droplets. Purified DNA particles were encapsulated in 18-pL droplets (λ ~ 0.1), and lacZ expression levels were measured by the accumulation of fluorescent product (fluorescein). To visualize DNA particles in droplets, the DNA stain (ethidium bromide) was used. Scale bars denote 50 μm.

To verify whether clonally-amplified DNA template condensed into DNA-Mg-PP_i_ particles support the in vitro transcription-translation reaction, we have amplified DNA carrying the lacZ gene by performing the MDA reaction in droplets as described above. After the MDA reaction, DNA particles were released from droplets and purified by brief digestion with restriction endonuclease followed by centrifugation (see Section 2.5). TEM analysis showed that DNA particles retained their condensed structure during purification process (Figure 6). The purified DNA-Mg-PP_i_ particles were then added to the IVTT reaction mixture in a 384-well plate and incubated for 3 h to allow lacZ protein synthesis to occur. As a control, we performed IVTT reactions having varying amounts of pIVEX2.2-lacZ plasmid ranging from 10^11^–10^8^ template copies in a 10-μL reaction volume. The enzymatic activity of the synthesized protein was then evaluated using a fluorogenic assay, which is based on hydrolysis of FDG (fluorescein-di-β-d-galactopyranoside) substrate to produce fluorescent product (fluorescein). Results presented in Figure 7 support the notion that DNA particles can serve as a template for protein synthesis in vitro, in agreement with previous work [22]. The yield of in vitro synthesized protein from only ~5 × 10^4^ DNA particles was approximately equal to the amount of protein produced from as many as ~10^9^–10^10^ copies of free DNA plasmid, thus suggesting that after purification, the single DNA particle retains ~10^4^–10^5^ copies of a functional gene. Alternatively, it could be that condensed DNA structures enhance the transcription-translation reaction, similarly to the DNA microgels reported previously [48]. To further validate the feasibility of DNA particles use as a biomaterial for the droplet-based assays, we co-encapsulated purified DNA-Mg-PP_i_ particles, the IVTT reaction mix and FDG substrate in 18-pL droplets using the 20 μm-deep microfluidics device depicted in Figure 2B. The collected IVT droplets were incubated at 37 °C for 1 h to allow lacZ gene expression to occur and emulsion imaged under fluorescent microscope. We used DNA particle dilution at λ ~ 0.1 to reduce the chance of having two particles in a droplet: in these conditions, ~89% of droplets would contain zero particles, 10% would contain one DNA particle and ~0.5% of all droplets would contain ≥2 DNA particles. As expected, the microscopic analysis revealed a droplet subpopulation (~10%) with bright fluorescent signal, thus indicating the reaction product formation due to the catalytic activity of β-galactosidase. The fluorescence signal corresponding to the enzymatic activity varied among droplets (coefficient of variation ~20%) and could be attributed to the differences of purified particle size (Figure 6) or varying amounts of DNA template incorporated into a particle. Nonetheless, considering early microfluidic systems [39] in which droplet reinjection and fusion steps were needed to perform protein synthesis from the amplified DNA template, this work significantly simplifies the use of in vitro expression assays and, therefore, could open new possibilities for directed evolution and synthetic biology fields. Finally, the better understanding of a mechanism by which amplified DNA is incorporated into macromolecular DNA-Mg-PP_i_ structures during the DNA amplification reaction may facilitate the improvement of whole genome amplification [49] and other isothermal nucleic acid amplification techniques [38]. 

## 4. Conclusions

An in vitro gene expression provides an important advantage for various screening applications as it enables the expression of proteins or enzymes that are toxic to the cells or that interfere with cell metabolic functions. The use of in vitro transcription-translation (IVTT) systems that rely on purified transcription and translation machinery components provide an attractive possibility to circumvent screening limitations imposed by living systems [50]. IVTT systems have been successfully applied for the expression of various recombinant proteins from the DNA templates [51,52]. Yet, to achieve sufficient amounts of in vitro synthesized protein, relatively high DNA template concentrations are needed (~10^9^ of DNA molecules per 10-μL reaction), and as a result, in vitro screening options for new catalytic activities in the synthetic gene libraries become largely restricted. Therefore, gene pre-amplification is often used before an in vitro expression step in order to circumvent the limited efficiency of IVTT systems [39,51]. For droplet microfluidics in particular, the DNA amplification by means of PCR can prove challenging, since long amplicons (>500 bp) are not amplified efficiently, and droplets’ integrity could be affected during thermocycling. Moreover, since biochemical conditions for the DNA amplification and IVTT reaction are different, the expression of proteins from the amplified DNA template would require precise fusion of individual droplets containing amplified DNA with droplets having IVTT reaction mix [39]. In this context, encapsulation of the DNA-Mg-PP_i_ particles carrying high amounts of clonally-amplified template provides a straightforward approach to increase the yield of in vitro expressed proteins in droplets without the need of performing complex, multi-step fluidic operations. Finally, the microfluidic approach reported here is likely to provide a promising method for the preparation of condensed DNA particles with encoded biological functionality for directed evolution, proteomics and synthetic biology applications.
